# Correlates of Nonanemic Iron Deficiency in Restless Legs Syndrome

**DOI:** 10.3389/fneur.2020.00298

**Published:** 2020-04-30

**Authors:** Xiao-Ying Zhu, Ting-Ting Wu, Hong-Ming Wang, Xuan Li, Ling-Yan Ni, Tian-Jiao Chen, Meng-Yao Qiu, Jun Shen, Te Liu, William G. Ondo, Yun-Cheng Wu

**Affiliations:** ^1^Department of Neurology, Shanghai General Hospital, Shanghai Jiao Tong University School of Medicine, Shanghai, China; ^2^Department of Clinical Laboratory Medicine, Shanghai General Hospital, Shanghai Jiao Tong University School of Medicine, Shanghai, China; ^3^Geriatric Laboratory, Shanghai Geriatric Institute of Chinese Medicine, Longhua Hospital, Shanghai University of Traditional Chinese Medicine, Shanghai, China; ^4^Department of Neurology, Methodist Neurological Institute, Weill Cornell Medical School, Houston, TX, United States

**Keywords:** restless legs syndrome, anemia, nonanemic, ferritin, iron deficiency

## Abstract

**Objective:** Iron deficiency anemia (IDA) is a well-known cause of secondary restless legs syndrome (RLS). Iron deficiency without anemia (IDNA) is insidious, and its association with RLS is less evaluated. We investigate prevalence and features of IDNA in a consecutive cohort of patients with RLS.

**Methods:** We included sequential primary RLS patients and RLS patients with IDA. We also recruited age- and gender-matched healthy controls. RLS mimics and other comorbidities were carefully excluded.

**Results:** One-hundred and ninety-six RLS patients without anemia, 26 RLS patients with IDA, and 63 controls were included. 42.3% of RLS patients without anemia had iron deficiency. Women were much more susceptible for IDNA with a relative risk of 5.51 (*p* < 0.0001). Women with IDNA and RLS had younger age both at interview and at RLS onset compared to women with RLS without iron deficiency (NID) (*P* < 0.01). IDNA RLS patients showed a tendency to higher risk of severe/very severe tiredness or sleepiness during the day as compared to NID RLS patients. Furthermore, IDNA RLS patients had longer duration of RLS (*P* < 0.01 in men, *P* < 0.05 in women) and younger age at onset (only in men, *P* < 0.05) compared to IDA RLS patients.

**Conclusion:** IDNA is frequent in RLS and iron deficiency may be severe despite a normal hemoglobin level. Women are at much higher risk for IDNA, and IDNA in women presents some specific clinical features. Features of IDNA RLS are different from IDA RLS. Regular screening of peripheral iron parameters even in patients with normal blood counts is recommended for timely optimal management.

## Introduction

Restless Legs Syndrome (RLS) is a common neurological disorder (1), and iron deficiency plays a key role in its pathogenesis (2, 3). Iron deficiency is common in RLS and iron deficiency anemia (IDA) is a well-known cause of secondary RLS (4, 5), which is associated with a six-fold increase in risk for RLS in the general population (6). Iron deficiency without anemia (IDNA, also nonanemic iron deficiency) is usually insidious, and recently attracted attention for its worldwide prevalence and challenge for diagnosis and management (7). Despite a clear relationship between clinical significance of RLS in IDA population (6), the prevalence and features of IDNA in RLS has not been systematically investigated. Whether IDNA represents a subgroup of RLS and whether IDNA is an early stage prior to IDA in RLS is unknown. In this study, we aim to investigate clinical features of RLS with IDNA compared to those without iron deficiency (NID, also non-iron deficiency) and to IDA patients in a consecutive cohort of Chinese RLS patients.

## Methods

### Inclusion and Exclusion of Patients

We continuously recruited patients from our RLS Specialist Clinic and Movement Disorders Clinic in Department of Neurology, Shanghai General Hospital, Shanghai Jiao Tong University School of Medicine between Jan. 2017 and Oct. 2018. RLS was diagnosed according to the 2014 International Restless Legs Syndrome Study Group (RLSSG) diagnostic criteria (1). Patients with other neurological disorders, poor cooperation, or cognitive impairment [Mini-Mental Status Examination (MMSE) score lower than 24 adjusted by education level] were excluded from the study. Age- and gender-matched healthy controls without anemia were recruited during the same period in the Health Examination Center in our hospital. Controls were free of neurological disease including RLS by face-to-face interview.

### Standard Protocol Approvals and Patient Consents

The study was approved by the Shanghai General Hospital Institutional Review Board and all patients and healthy controls included in the study gave written consent.

### Examination Program

RLS patients were evaluated as we previously described (8). Briefly, we interviewed patients face-to-face in our outpatient clinic. Patients considered positive for RLS were diagnosed by two neurologists specialized in RLS. We recorded demographic information, history, symptoms, medications, general neurologic and medical examinations, and MMSE for all recruited patients. A semi-structured questionnaire was further assessed, which included age of RLS onset, RLS family history (familiarity defined as “first-degree relatives”), type and topography of sensations, and if RLS symptoms correlated with seasonal variation. RLS severity for 1 week prior to the interview was assessed using the International RLS rating scale (IRLSRS) (9). Examinations such as vascular ultrasound, nerve conduction velocities, electromyography, etc. were performed if clinically indicated to exclude possible RLS mimics like peripheral polyneuropathies, venous stasis, vascular claudication, rheumatoid arthritis, and so on. Subjects with medication history of neuroleptics or other medication that the authors considered possibly related to RLS in the preceding three months were also excluded. We excluded those with notable comorbidities that were associated with secondary RLS like chronic renal failure, pregnancy, Parkinson's disease, peripheral neuropathy, stroke, and ataxia. Blood tests for hemoglobin and peripheral iron status including serum ferritin, iron, transferrin, and total iron-binding capacity (TIBC) were assessed. Transferrin saturation (TSAT) was calculated as serum iron/TIBC × 100. Normal hemoglobin level was defined as women ≥ 113 g/L and men ≥ 131 g/L according to the normal value in our hospital. IDNA is defined as serum ferritin <75 μg/L (or ferritin ≥ 75 μg/L but TSAT <20%) with normal hemoglobin level (10, 11). IDA is diagnosed when hemoglobin <113 g/L (women) or <131 g/L (men), as well as serum ferritin <75 μg/L (or serum ferritin ≥ 75 μg/L but TSAT <20%).

### Statistics

Data were analyzed using SPSS 21.0 for Windows (IBM Co., USA). All data are presented as means ± standard deviations. Since most of the variables were not normally distributed, the Kruskal-Wallis test was used for single variable comparisons among three groups, and *post-hoc* Mann–Whitney when *P* < 0.05. Differences in proportions were analyzed by Pearson's chi-squared or Fisher's exact test when appropriate. *P* < 0.05 were considered significant. Considering the potential effects of drug therapy on RLS symptom and other features, we calculated all the data twice in the total RLS patients and in RLS patients without medication, respectively.

## Results

We continuously enrolled a total of 196 primary RLS patients without anemia (IDNA RLS and NID RLS) in this study, including 66 male patients (33.7%) and 130 female patients (66.3%). The average age was 53.0 ± 13.5 years old. 65.8% (129/196) of the RLS patients were not taking medications for RLS (109 drug-naïve, and 20 drug-free for more than 2 weeks at interview). We also included 26 RLS patients with IDA (22 drug-naïve). We excluded five anemic RLS patients (Hb <113 g/L for women and Hb <131 g/L for men) who did not have evidence of iron deficiency (serum ferritin ≥ 75 μg/L as well as TIBC ≥ 20%, mean serum ferritin as 236.1 ± 119.3 μg/L). None of the RLS patients with IDA had any signs of infection, and causes of IDA RLS were listed (Table 1). In addition, we collected 63 healthy controls subjects including 22 male subjects (34.9%) and 41 female subjects (65.1%), and the average age of the control group was 51.0 ± 12.0 years old (the average age of 51.0 ± 10.4 and 51.0 ± 12.8 for male and female, respectively). There were no significant differences in both gender (RLS vs. control: 66.3 vs. 65.1% for female, *P* > 0.05) and age (RLS vs. control: 53.0 ± 13.5 vs. 51.0 ± 12.0, *P* > 0.05) between RLS patients and controls. Detailed demographic information of patients with RLS in each group are shown (Table 2).

**Table 1:** Possible causes of iron deficiency in anemic RLS patients.

	**Male**	**Female**	**Total**
Ulcer hemorrhage	1	1	2
Hemorrhoid hemorrhage	2	0	2
Menometrorrhagia	/	10	10
Malnutrition	4	6	10
Unknown causes	0	2	2

*RLS, restless legs syndrome*.

**Table 2:** Demographic information and clinical features of RLS patients with IDNA, NID, and IDA.

	**RLS with IDNA (*n* = 83)**	**RLS with NID (*n* = 113)**	**RLS with IDA (*n* = 26)**	***P*-value**
Female, *n* (%)	76 (91.6%)	54 (47.8%)	19 (73.1%)	***X*****2** **=** **42.029**, ***P*** **<** 0.0001, ab*, ac, bc
Age (year)	48.2 ± 14.8	56.5 ± 11.4	51.2 ± 13.8	***H*** **=** **21.645**, ***P*** **<** 0.0001, ab*, bc
Male	53.0 ± 12.8	53.9 ± 11.8	61.9 ± 10.1	*H* = 3.625, *P* = 0.163
Female	47.7 ± 14.9	59.4 ± 10.3	47.3 ± 13.0	***H*** **=** **28.477**, ***P*** **<** 0.0001, ab*, bc*
Shanghai residents, *n* (%)	20 (24.1%)	31 (27.4%)	6 (23.1%)	*X*2 = 0.383, *P* = 0.826
Non-Shanghai residents, *n* (%)	63 (75.9%)	82 (72.6%)	20 (76.9%)	*X*2 = 0.383, *P* = 0.826
East China (non-Shanghai)	44 (53.0%)	64 (56.6%)	17 (65.4%)	*X*2 = 1.242, *P* = 0.537
Northeast China	5 (6.0%)	5 (4.4%)	0 (0%)	*X*2 = 1.161, *P* = 0.595
North China	4 (4.8%)	1 (0.9%)	0 (0%)	*X*2 = 3.013, *P* = 0.244
Central China	5 (6.0%)	3 (2.7%)	2 (7.7%)	*X*2 = 2.385, *P* = 0.251
South China	1 (1.2%)	2 (1.8%)	0 (0%)	*X*2 = 0.420, *P* = 1.000
Northwest China	2 (2.4%)	3 (2.7%)	1 (3.8%)	*X*2 = 0.628, *P* = 0.720
Southwest China	2 (2.4%)	4 (3.5%)	0 (0%)	*X*2 = 0.516, *P* = 1.000
Hyperlipidemia, *n* (%)	15 (18.1%)	26 (23.0%)	2 (7.7%)	*X*2 = 3.318, *P* = 0.19
Hypertension, *n* (%)	10 (12.0%)	21 (18.6%)	3 (11.5%)	*X*2 = 1.918, *P* = 0.383
Diabetes, *n* (%)	5 (6.0%)	9 (8.0%)	5 (19.2%)	*X*2 = 3.689, *P* = 0.158
Anemia, *n* (%)	0 (0%)	0 (0%)	26 (100%)	*X*2 = 149.23, *P* < 0.0001, ac*, bc*
Without medication for RLS, *n* (%)	60 (72.3%)	69 (61.1%)	22 (84.6%)	*X*2 = 6.502, *P* = 0.039, bc
On medication for RLS, *n* (%)	23 (27.7%)	44 (38.9%)	4 (15.4%)	*X*2 = 6.502, *P* = 0.039, bc
Dopaminergics	21 (91.3%)	40 (90.9%)	4 (100.0%)	*X*2 = 0.255, *P* = 1
Gabapentin enacarbil	6 (26.1%)	4 (9.1%)	0 (0%)	*X*2 = 3.539, *P* = 0.156
Iron supplementation	0 (0%)	0 (0%)	1 (25.0%)	*X*2 = 6.635, *P* = 0.056
Others	4 (17.4%)	10 (22.7%)	0 (0%)	*X*2 = 0.755, *P* = 0.697
Age at RLS onset (year)	33.6 ± 15.6	40.8 ± 14.8	44.1 ± 16.8	***H*** **=** **13.603**, ***P*** **=** 0.001, ab*, ac*
Male	43.7 ± 14.9	40.9 ± 14.1	59.0 ± 10.1	***H*** **=** **10.299**, ***P*** **=** 0.006, ac, bc*
Female	32.7 ± 15.4	40.7 ± 15.6	38.6 ± 15.5	***H*** **=** **9.126**, ***P*** **=** 0.01, ab*
Duration of RLS (year)	14.5 ± 11.7	15.8 ± 12.1	7.1 ± 7.0	***H*** **=** **13.886**, ***P*** **=** 0.001, ac*, bc*
Male	9.2 ± 6.5	13.2 ± 10.4	2.9 ± 1.1	***H*** **=** **9.109**, ***P*** **=** 0.011, ac*, bc*
Female	15.0 ± 12.0	18.7 ± 13.1	8.6 ± 7.6	***H*** **=** **10.308**, ***P*** **=** 0.006, ac, bc*
RLS family history, *n* (%positive)	28 (33.7%)	37 (32.7%)	6 (23.1%)	*X*2 = 1.095, *P* = 0.578
Male	3 (42.9%)	17 (28.8%)	2 (28.6%)	*X*2 = 0.824, *P* = 0.728
Female	25 (32.9%)	20 (37.0%)	4 (21.1%)	*X*2 = 1.627, *P* = 0.443
IRLSRS	24.1 ± 7.8	24.1 ± 6.5	25.7 ± 6.8	*H* = 1.059, *P* = 0.589
Male	22.6 ± 8.6	23.4 ± 6.9	23.9 ± 5.1	*H* = 0.123, *P* = 0.94
Female	24.3 ± 7.8	24.9 ± 6.1	26.4 ± 3.8	*H* = 0.39, *P* = 0.823
IRLSRS, severe to very severe (21–40), *n* (%)	60 (72.3%)	87 (77.0%)	23 (88.5%)	*H* = 2.909, *P* = 0.234
Severe sleep disturbance due to RLS (IRLSRS item 4 ≥ 3), *n* (%)	56 (69.1%)	79 (69.9%)	22 (84.6%)	*X*2 = 2.884, *P* = 0.237
Severe tiredness or sleepiness during the day due to RLS (IRLSRS item 5 ≥ 3), *n* (%)	27 (32.5%)	20 (17.7%)	8 (30.8%)	***X*****2** **=** **6.215**, ***P*** **=** **0.045, ab**
Impact on daily affairs due to RLS (IRLSRS item 9 ≥ 3), *n* (%)	12 (14.5%)	15 (13.3%)	3 (11.5%)	*X*2 = 0.14, *P* = 0.961
Severe mood disturbance due to RLS (IRLSRS item 10 ≥ 3), *n* (%)	23 (28.4%)	24 (21.2%)	7 (26.9%)	*X*2 = 1.197, *P* = 0.55
Chronic-persistent RLS, *n* (%)	73 (88.0%)	105 (92.9%)	26 (100%)	*X*2 = 3.844, *P* = 0.148
Unilateral or unilateral dominant of RLS, *n* (%)	36 (43.4%)	44 (38.9%)	9 (34.6%)	*X*2 = 0.759, *P* = 0.684
Strictly unilateral RLS, *n* (%)	4 (4.8%)	7 (6.2%)	2 (7.7%)	*X*2 = 0.598, *P* = 0.714
Extra body parts involvement beyond legs, *n* (%)	20 (24.1%)	21 (18.6%)	4 (15.4%)	*X*2 = 1.335, *P* = 0.513
Seasonal fluctuation	27 (32.5%)	38 (33.6%)	9 (34.6%)	*X*2 = 0.048, *P* = 0.976
With worsening in summer, *n* (%)	14 (16.9%)	16 (14.2%)	4 (15.4%)	*X*2 = 0.347, *P* = 0.897

Kruskal-Wallis test for comparison of continuous variables, post-hoc Mann-Whitney when p < 0.05, Pearson's chi-squared or Fisher's exact test for categorical variables. Iron deficiency was defined as ferritin level <75 μg/L, or ferritin ≥ 75 μg/L, however, TSAT <20%.

IDA, Iron deficiency anemia; IDNA, Iron deficiency without anemia; NID, non-iron deficient; IRLSRS, International Restless Legs Syndrome Rating Scale; RLS, restless legs syndrome.

ac: IDNA vs. IDA at p < 0.05.

ac^*^: IDNA vs. IDA at p < 0.01.

bc: NID vs. IDA at p < 0.05.

bc^*^: NID vs. IDA at p < 0.01.

ab: IDNA vs. NID at p < 0.05.

ab^*^: IDNA vs. NID at p < 0.01.

*p < 0.05 were emphasized in bold*.

### Frequency of IDNA in RLS Patients

Of the 196 RLS patients (130 women, 66 men) without anemia or other comorbidities, 83 (42.3%) had iron deficiency. Prevalence of IDNA in women (58.5%, 76/130) is much higher than in men (10.6%, 7/66) in this cohort of RLS patients without anemia (Table 2), with a relative risk of 5.51 (95% confidence interval [CI] 2.70–11.27, *p* < 0.0001).

### Clinical Features of RLS Patients With IDNA Compared to NID

**Figure 1:**
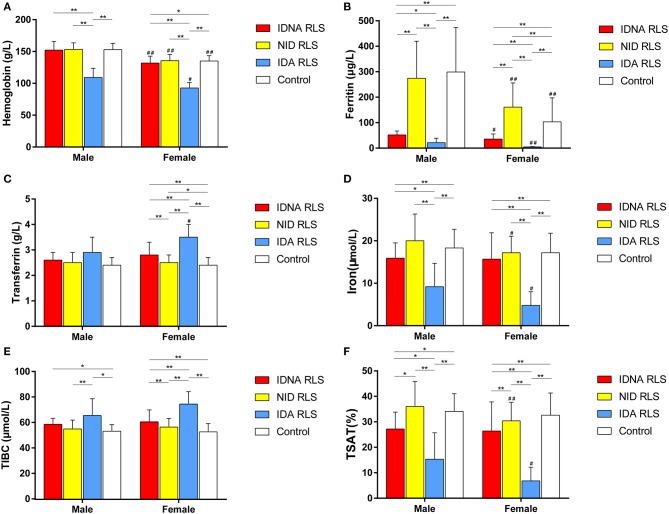
The hematologic and iron parameters by group and gender **(A)** Hemoglobin. **(B)** Ferritin. **(C)** Transferrin. **(D)** Iron. **(E)** TIBC. **(F)** TSAT. **P < 0.01, *P < 0.05, Kruskal-Wallis test and *post-hoc* Mann-Whitney test. Data compared between IDNA RLS (*n* = 83), NID RLS (*n* = 113), IDA RLS (*n* = 26), and controls (*n* = 63). ^##^*P* < 0.01, ^#^*P* < 0.05, Mann-Whitney *U*-test. Data compared between male and female in each group of IDNA RLS, NID RLS, IDA RLS, and controls. Iron deficiency was defined as ferritin level <75 μg/L, or ferritin 75 μg/L, however TSAT <20%. IDA, Iron deficiency anemia; IDNA, Iron deficiency without anemia; NID, non-iron deficient; RLS, restless legs syndrome; TIBC, total iron-binding capacity; TSAT, transferrin saturation.

In female RLS patients without anemia, the IDNA group had a younger age both at interview and at RLS onset when compared to the NID group (*P* < 0.01; Table 2). Although there was no difference on severity of RLS by IRLSRS, we found more patients with severe/very severe tiredness or sleepiness during the day in the IDNA group when compared to the NID group (32.5 vs. 17.7%, *P* < 0.05; Table 2). There was no difference regarding family history, severity, laterality, location, and seasonal fluctuation of RLS between IDNA and NID group, neither in men nor in women. The IDNA RLS group presented lower serum ferritin (male and female, *P* < 0.0001) as well as higher transferrin and TIBC values (female, *P* < 0.0001) as compared to the NID RLS group (Figure 1, Table 3).

**Table 3:** Hematologic data of RLS patients with IDNA, NID, IDA, and controls.

	**RLS with IDNA (*n* = 83)**	**RLS with NID (*n* = 113)**	**RLS with IDA (*n* = 26)**	**Controls (*n* = 63)**	***P*-value**
Hemoglobin (g/L)	133.6 ± 12.4	144.8 ± 13.4	97.2 ± 12.6	141.3 ± 12.4	***H*** **=** **96.508**, ***P*** **<** 0.0001, ab*, ac*, bc*, ad*, cd*
Male	152.3 ± 13.5	153.4 ± 10.2	109.3 ± 14.5	152.9 ± 10.0	***H*** **=** **19.100**, ***P*** **<** 0.0001, ac*, bc*, cd*
Female	131.9 ± 10.8	135.6 ± 10.0	92.7 ± 8.5	135.0 ± 8.4	***H*** **=** **56.793**, ***P*** **<** 0.0001, ac*, bc*, ad, cd*
Ferritin (μg/L)	37.1 ± 20.5*	220.2 ± 136.0	8.8 ± 11.2*	171.6 ± 157.9*	***H*** **=** **179.496**, ***P*** **<** 0.0001, ab*, ac*,bc*, ad*, bd*, cd*
Male	51.9 ± 15.4*	274.2 ± 145.7	21.4 ± 17.7*	299.1 ± 174.9	***H*** **=** **31.003**, ***P*** **<** 0.0001, ab*, ac, bc*, ad*, cd*
Female	35.6 ± 20.2*	161.1 ± 95.1	4.6 ± 1.4*	103.2 ± 94.1*	***H*** **=** **126.278**, ***P*** **<** 0.0001, ab*, ac*, bc*, ad*, bd*, cd*
Transferrin (g/L)	2.8 ± 0.5	2.5 ± 0.3	3.3 ± 0.6	2.4 ± 0.3	***H*** **=** **62.219**, ***P*** **<** 0.0001, ab*, ac*, bc*, ad*, bd, cd*
Male	2.6 ± 0.3	2.5 ± 0.4	2.9 ± 0.6	2.4 ± 0.3	*H* = 7.323, *P* = 0.062
Female	2.8 ± 0.5	2.5 ± 0.3	3.5 ± 0.5	2.4 ± 0.3	***H*** **=** **52.619**, ***P*** **<** 0.0001, ab*, ac*, bc*, ad*, bd, cd*
Iron (μmol/L)	15.7 ± 6.0	18.6 ± 5.4	6.0 ± 4.3	17.6 ± 4.5	***H*** **=** **66.980**, ***P*** **<** 0.0001, ab*, ac*, bc*, ad*, cd*
Male	15.9 ± 3.6	20.0 ± 6.3	9.2 ± 5.5	18.3 ± 4.4	***H*** **=** **15.895**, ***P*** **=** 0.001, ac, bc*, ad*, cd*
Female	15.7 ± 6.2	17.2 ± 3.9	4.8 ± 3.2	17.2 ± 4.6	***H*** **=** **52.619**, ***P*** **<** 0.0001, ac*, bc*, ad*, cd*
TIBC (μmol/L)	60.6 ± 8.8	56.0 ± 6.5	72.4 ± 10.9	53.2 ± 5.7	***H*** **=** **68.399**, ***P*** **<** 0.0001, ab*, ac*, bc*
Male	58.8 ± 4.5	55.3 ± 6.6	65.8 ± 12.8	53.5 ± 4.8	***H*** **=** **9.955**, ***P*** **=** 0.019, bc*, ad, cd
Female	60.8 ± 9.1	56.8 ± 6.3	74.8 ± 9.4	53.0 ± 6.1	***H*** **=** **55.789**, ***P*** **<** 0.0001, ab*, ac*, bc*, ad*, bd*, cd*
TSAT	26.4 ± 11.0%	33.3 ± 9.1%	9.1 ± 7.9%	33.2 ± 8.1%	***H*** **=** **85.350**, ***P*** **<** 0.0001, ab*, ac*, bc*, ad*, cd*
Male	27.2 ± 6.6%	36.0 ± 9.8%	15.3 ± 10.4%	34.1 ± 6.9%	***H*** **=** **19.827**, ***P*** **<** 0.0001, ab, ac, bc*, ad, cd*
Female	26.4 ± 11.4%	30.4 ± 7.3%	6.8 ± 5.4%	32.6 ± 8.7%	***H*** **=** **60.660**, ***P*** **<** 0.0001, ab*, ac*, bc*, ad*, cd*

Kruskal-Wallis test for comparison of continuous variables, post-hoc Mann-Whitney when p < 0.05, Pearson's chi-squared or Fisher's exact test for categorical variables. Iron deficiency was defined as ferritin level <75 μg/L, or ferritin ≥ 75 μg/L, however, TSAT <20% in patients with RLS.

IDA, Iron deficiency anemia; IDNA, Iron deficiency without anemia; NID, non-iron deficient; RLS, restless legs syndrome; TIBC, total iron-binding capacity; TSAT, transferrin saturation.

^*^Values of serum ferritin ≥ 75 μg/L (all with TSAT <20%) were excluded for statistics in the IDNA (n = 4), IDA (n = 2), and control group (n = 1) since these ferritin values may be ostensible due to inflammatory or other conditions.

ac: IDNA vs. IDA at p < 0.05; ac^*^: IDNA vs. IDA at p < 0.01.

bc: NID vs. IDA at p < 0.05; bc^*^: NID vs. IDA at p < 0.01.

ab: IDNA vs. NID at p < 0.05; ab^*^: IDNA vs. NID at p < 0.01.

ad: IDNA vs. control at p < 0.05; ad^*^: IDNA vs. control at p < 0.01.

bd: NID vs. control at p < 0.05; bd^*^: NID vs. control at p < 0.01.

cd: IDA vs. control at p < 0.05; cd^*^: IDA vs. control at p < 0.01.

*p < 0.05 were emphasized in bold*.

After excluding RLS patients undergoing medications for RLS, the IDNA RLS group still showed higher rates of daytime severe/very severe tiredness/sleepiness as compared to the NID RLS group, though not a significant level (35 vs. 18.8%, *P* = 0.078). Other features between IDNA RLS and NID RLS were similar (Supplementary Table 1).

### Clinical Features of RLS Patients With IDNA Compared to IDA

The IDA group had more severely disturbed serum iron metabolic parameters than the IDNA group, including lower ferritin (male, *P* < 0.05; female, *P* < 0.0001), higher transferrin (female, *P* < 0.0001), lower iron (male, *P* < 0.05; female, *P* < 0.0001), higher TIBC (female, *P* < 0.0001), and lower TSAT (male, *P* < 0.05; female, *P* < 0.0001) values in serum (Figure 1, Table 3). IDNA RLS had longer disease duration (*P* < 0.01 in men, *P* < 0.05 in women) and younger age at onset (only significant in men with *P* < 0.05) than IDA RLS. There were no differences regarding positive family history, severity (IRLSRS), laterality, location, and seasonal fluctuation of RLS between IDNA RLS and IDA RLS, either in men or in women RLS patients (Table 2). The results were similar after excluding RLS patients with medications for RLS (Supplementary Table 1).

### Hematological Features in Control Subjects

Diagnostic criteria for iron deficiency are different between the general population and RLS patients (as discussion below). According to our hospital's cut-off value for iron deficiency in the general population (20 μg/L), we found none of the male subjects in the control group had iron deficiency, while four female control subjects showed iron deficiency (two subjects with serum ferritin <20 μg/L, and two subjects with ferritin ≥ 20 μg/L but TSAT <20%). There was no significant difference of prevalence of iron deficiency in controls between men (0%, 0/22) and women (9.8%, 4/41; *P* = 0.288). The hematological tests on hemoglobin and iron parameters in the control group were most similar with the NID RLS group as compared to the IDNA or IDA group (Figure 1, Table 3).

## Discussion

The pathophysiology of primary and secondary RLS is incompletely understood. Brain iron deficiency has a fundamental role in RLS pathogenesis, and conditions characterized by systemic iron deficiency, such as anemia and pregnancy, are associated with a higher prevalence of RLS symptoms (2, 3, 5, 6). IDA is a recognized comorbidity associated with secondary RLS (4–6, 12). IDNA is more insidious, and its features are less known in RLS patients.

### Clinical Features of IDNA in RLS

In general, females are more susceptible to RLS (1, 13, 14). Women of childbearing age are also more iron deficient than men in the general population (15). In this study on RLS patients without anemia, we also found a significant gender-dependent discrimination. Female RLS patients are at much higher risk for IDNA than male RLS patients (relative risk of 5.51, *p* < 0.0001). Fewer male RLS patients suffered from iron deficiency, either with or without anemia. In controls, although without a statistical significance, we found 9.8% of the female subjects suffered from iron deficiency while none of the male subjects did. Iron deficiency is uncommon in the male gender, so if male RLS patients have iron deficiency, additional enquires should be made to evaluate for secondary causes such as chronic bleeding and malnutrition due to gastrointestinal disorders.

When compared to the “normal” NID RLS group, the IDNA RLS group showed an earlier RLS onset age and a younger age at interview in women, but not in men (Table 2). In an earlier study, we found early onset RLS demonstrated more positive RLS family history and more severely disturbed peripheral iron parameters (lower serum ferritin, and higher serum transferrin and TIBC values) when compared to late onset RLS (8). Recently, we found IDNA in RLS predicts some genetic factors when compared to NID in RLS (unpublished data). A potential subtype in association with genetic background, iron deficiency, and early onset RLS deserves further investigation. Fatigue, weakness, difficulty in concentrating, decreased memory, as well as poor work performance are nonspecific symptoms that often associated with iron deficiency (7, 16). Allen et al. (6) previously reported IDA sufferers with RLS showed more tiredness, poorer sleep quality, and decreased daytime energy compared to IDA patients without RLS. We found that RLS patients with IDNA were also more likely to suffer from manifestations of severe/very severe tiredness or sleepiness during the day due to RLS compared to RLS patients with NID (cut-off point at 75 μg/L, 32.5 vs. 17.7%, *P* < 0.05, Table 2). When analyzed again only in RLS patients without medication, the IDNA RLS patients still showed a trend of more susceptible to daytime severe/very severe tiredness or sleepiness, although not reaching a statistical significance (35 vs. 18.8%, *P* = 0.078, Supplementary Table 1). No difference of severe/very severe daytime tiredness or sleepiness was found between IDNA RLS and IDA RLS in this study (Table 2). Depleted iron store, along a continuum from IDA to IDNA, is associated with decreased activity of iron dependent enzymes, reduced cellular oxidative capacity, as well as decreased energy efficiency (17–19). When we used a stricter definition for IDNA (serum ferritin cut-off value set at 50 μg/L), we still found more IDNA RLS patients suffered from severe/very severe tiredness or sleepiness during the day as compared to NID RLS patients (32.3 vs. 19.8%), however this was not significant (*P* > 0.05; Supplementary Table 2). Periodic leg movement in sleep (PLMS) is closely related to RLS, and locus within the BTBD9 gene is correlated to risk of both PLMS and RLS (20, 21). Notably, we only evaluated sleep quality using the IRLSRS subitem 4, and we didn't perform a detailed sleep status assessment using the polysomnography (PSG) measure. Consequently, data of PLMS and other sleep related parameters were unavailable, which may significantly contribute to daytime tiredness or sleepiness. Therefore, the association between daytime tiredness/sleepiness and IDNA needs further investigation. We didn't find significant difference in severity of RLS between the IDNA and the NID group. Peripheral iron deficiency will increase risk for RLS prevalence in previous clinical studies (6). Reduced serum iron concentrations globally decrease brain iron levels, and iron treatment benefits RLS patients with low levels of peripheral iron (10). Nevertheless, numerous RLS patients without systemic iron deficiency still display a brain-specific deficit in iron (22). Studies in rodents reported serum iron status was not correlated with regional brain iron levels, therefore may be limitedly related to features of RLS (23, 24).

### Is IDNA an Early Stage Prior to IDA in RLS?

Iron deficiency can be classified into two levels, IDNA and IDA, according to the hemoglobin measurement value. In this study, RLS patients with IDA had the lowest hemoglobin, ferritin, and iron levels, as well as the highest transferrin and TIBC values as compared to IDNA RLS and NID RLS (Figure 1, Table 3). Although there were no significant differences on hemoglobin levels between IDNA RLS and NID RLS (both in male and female RLS patients), serum hemoglobin in IDNA RLS patients were lower compared to controls (only in female, *P* < 0.05, Figure 1, Table 3). In addition, values of iron parameters in the IDNA RLS group were between those of the NID RLS or the control group and the IDA RLS group (Figure 1, Table 3). These results are consistent with the viewpoint of IDNA is a milder iron deficiency prior to the development of anemia. Of note, serum iron parameters are considered reflecting the peripheral iron status mainly in the erythrons (25), while iron storage in other organs (such as liver, brain, etc.) need validated in IDNA subjects.

Nevertheless, causes and features of IDNA RLS and IDA RLS are not entirely similar. In this study, most RLS patients with IDA had notable comorbidities associated with malnutrition or chronic excessive loss of blood, including alimentary tract hemorrhage, hemorrhoid hemorrhage, and menorrhagia (Table 1), which is in line with the previous study (12). It is worth noting that menorrhagia is a common cause of IDA in women of reproductive age, which can be subsequent to uterine fibroids, adenomyosis, and other gynecological disorders. Malnutrition can be secondary to gastrointestinal disorders, vegetarian diet, and misconceptions regarding self-body image in young women. Thus, iron deficiency in majority of IDA RLS patients in this study are “absolute iron deficiency.” In contrast, RLS patients with IDNA usually lack notable comorbidities. Causes of IDNA RLS are insidious and IDNA in RLS generally belongs to the scope of primary RLS (4) associated with “functional iron deficiency.” However, we can't exclude any insidious secondary causes in IDNA RLS patients, which may lead to inadequate supply of iron in the body.

Compared to RLS with IDNA, patients with IDA had an older age of RLS onset, as well as shorter duration of RLS in both male and female (more significant in male; Table 2), which was in accordance with the viewpoint that secondary RLS usually has an older onset age (26). According to our experience, these IDA RLS patients with notable causes (such as acute blood loss) usually demonstrate shorter disease duration and more rapid recovery or relief when treatable comorbid disorders are corrected and iron status restored. They usually don't require long-term RLS therapy, which are somewhat different from primary RLS patients. Future longitudinal studies are necessary to confirm the treatment effects of iron supplement in RLS patients with IDA. Other studies on comorbid RLS of pregnancy (27) and uremia (28) reported improvement after delivery and renal transplant, suggesting changes in associated metabolic factors may trigger RLS in susceptible subjects (5).

Therefore, there are heterogeneity in both IDA RLS and IDNA RLS. Although IDNA is generally considered an early stage prior to anemia (7), IDNA RLS is possibly not simply a prodromal stage prior to IDA RLS.

### Accessing and Diagnosing IDNA in RLS

Although bone marrow biopsy is regarded the best indicator for iron status, it is seldom performed due to potential risks of infection or bleeding at the biopsy site (29). The most widely used laboratory indicators of iron status are ferritin, which reflects either tissue iron stores or the adequacy of iron readily available for erythropoiesis (30). Although serum ferritin is a sensitive marker for evaluating total body iron store, other conditions such as inflammatory status, age, and renal function may be confounders, increasing serum ferritin levels (31). Ferritin and transferrin are also acute-phase reactants, and ferritin level could be increased and transferrin levels be decreased under an inflammatory condition (32). Therefore, even serum ferritin ≥ 75 μg/L and TSAT <20% could still have actual iron deficiency (11, 33).

A serum ferritin concentration cut off point of <30 μg/L is most sensitive and specific to identify potential iron deficiency in the general population (17, 18), and IDNA is classified as having normal hemoglobin levels with decreased serum ferritin levels (<20 μg/L) (34, 35). Clinical laboratories usually set the lower limit of the reference range for serum ferritin at 10–20 μg/L in the common population (36) (the lower limit is set at 20 μg/L in our hospital). Significantly higher threshold levels for serum ferritin are usually used to define iron deficiency in chronic disorders such as heart failure and chronic kidney disease (7, 17). The assessment criteria to define iron deficiency in RLS is also different from that in healthy controls, although the exact cut-off value for ferritin to signify IDNA in RLS has not yet reached consensus. Recent studies suggest RLS patients should be considered iron deficient when their ferritin concentration is below 75 μg/L (10, 11, 27). However, earlier studies mostly determined ferritin concentration under 50 μg/L as “low iron status” in RLS (37, 38). Since no data on descriptions of different cut off point are available, we compared features of IDNA RLS and NID RLS using both 75 and 50 μg/L as the cut-off values of ferritin for iron deficiency. We found that when using 50 μg/L as the lower board line of ferritin, 33.2% (65/196) of the RLS patients meet the criteria of IDNA. Results were quite similar for the two used thresholds, except that increased severe/very severe tiredness or sleepiness during the day in IDNA RLS compared to NID RLS was not significant when the cut-off value of ferritin was set at 50 μg/L (Table 2, Supplementary Table 2).

Our study had some limitations: First, this was a cross-sectional study and lack of longitudinal data made it hard to identify the evolution of RLS symptoms and effects of treatment. Furthermore, there may be a recall bias. Second, this was a single center study as about a quarter of the patients were local Shanghai residents and the majority of the patients came from East China. Considering a vast territory in China and possible various environmental factors, there may be a selection bias. Third, we didn't perform PSG measurement to evaluate detailed sleep status in RLS patients and controls. Fourth, about one-third of RLS patients in this study were on dopaminergics and/or gabapentin enacarbil therapy (only one patient was receiving iron supplementary therapy), which may influence clinical features. However, we calculated all the data again in RLS patients without medication and drew the same conclusion. That being said, our study does have some advantages. We studied in detail the clinical spectrum of RLS and excluded other comorbidities or RLS mimics. We interviewed each patient face-to-face, and we carefully excluded those atypical RLS patients during follow-up. There have been limited studies on features of IDNA in RLS, we explored detailed clinical characteristics of IDNA in consecutive Chinese RLS patients.

## Conclusion

IDNA is frequent in patients with RLS especially in women and has some special clinical features. IDNA had younger age both at interview and at RLS onset when compared to NID in female RLS patients. IDNA is not very common in male RLS patients and men with iron deficiency should be evaluated for comorbid disorders. Features of RLS patients with IDNA are different from RLS with IDA. RLS with IDNA represents an important and overlooked public health problem that deserves more clinical attention and should be addressed in the treatment strategy. Regular screening of peripheral iron parameters even in patients with normal blood count is recommended for timely appropriate therapy.

## Data Availability Statement

The datasets generated for this study are available on request to the corresponding author.

## Ethics Statement

The studies involving human participants were reviewed and approved by the Shanghai General Hospital Institutional Review Board. The patients/participants provided their written informed consent to participate in this study.

## Author Contributions

X-YZ conception/design/execution of the project/drafting the manuscript. T-TW: execution of the project/collection and interpretation of data/drafting the manuscript. H-MW and JS: laboratory measurement. XL, L-YN, T-JC, and M-YQ: execution of the project/collection and interpretation of data. TL and WO: critical revision of the manuscript for important intellectual content. Y-CW: conception/design/supervision of the project, critical revision of the manuscript for important intellectual content. All authors: manuscript preparation.

## Conflict of Interest

The authors declare that the research was conducted in the absence of any commercial or financial relationships that could be construed as a potential conflict of interest.

## Abbreviations

CI: confidence interval
IRLSSG: International Restless Legs Study Group
MMSE: Mini-Mental State Examination
NID: without iron deficiency
RLS: restless legs syndrome
IDA: iron deficiency anemia
IDNA: iron deficiency without anemia
IRLSRS: International Restless Legs Syndrome Rating Scale
TIBC: total iron binding capacity
TSAT: transferrin saturation.
