# Supplementary material for: Correlates of Nonanemic Iron Deficiency in Restless Legs Syndrome
Source: Front Neurol. 2020 Apr 30;11:298. doi: 10.3389/fneur.2020.00298 (PMC7205016; doi:10.3389/fneur.2020.00298)
Supplement: Supplementary file 1 [file Table_1.DOCX]

**Supplementary table 1: Demographic information, clinical features and laboratory tests in drug naïve/drug free RLS patients with IDNA, NID and IDA**

|  | **RLS with IDNA**  **(n=60)** | **RLS with NID**  **(n=69)** | **RLS with IDA**  **(n=22)** | **P value** |
| --- | --- | --- | --- | --- |
| **Female, n (%)** | 55 (91.7%) | 40 (58.0%) | 17 (77.3%) | **X2=19.15, P<0.0001, ab*** |
| **Age (yr)** | 44.6±12.7 | 55.4±12.4 | 50.1±14.5 | **H=22.458, P<0.0001, ab*, bc** |
| **male** | 47.8±11.3 | 49.2±11.4 | 61.2±12.3 | H=3.943, P=0.139 |
| **female** | 44.3±12.9 | 59.9±11.2 | 46.9±13.7 | **H=32.022, P<0.0001, ab*, bc*** |
| **Hyperlipidemia, n (%)** | 10 (16.7%) | 15 (21.7%) | 2 (9.1%) | X2=1.71, P=0.421 |
| **Hypertension, n (%)** | 7 (11.7%) | 13 (18.8%) | 3 (13.6%) | X2=1.273, P=0.582 |
| **Diabetes, n (%)** | 4 (6.7%) | 4 (5.8%) | 3 (13.6%) | X2=1.728, P=0.479 |
| **Anemia, n (%)** | 0 (0%) | 0 (0%) | 22 (100%) | **X2=114.691, P<0.0001, ac*, bc*** |
| **Age at RLS onset (yr)** | 32.5±14.9 | 38.1± 16.4 | 42.3±17.5 | **H=6.138, P=0.046, ac** |
| **male** | 39.6±14.5 | 36.0± 15.4 | 58.2±12.3 | **H=7.240, P=0.027, ac, bc*** |
| **female** | 31.8±14.9 | 39.6± 17.2 | 37.6±16.1 | H=5.117, P=0.077 |
| **Duration of RLS (yr)** | 12.1±8.7 | 17.4± 12.8 | 7.8±7.4 | **H=13.629, P=0.001, ab, ac, bc*** |
| **male** | 8.1±4.2 | 13.4± 10.5 | 3.0±0.7 | **H=6.743, P=0.034, ac, bc** |
| **female** | 12.4±9.0 | 20.3± 13.6 | 9.2±7.9 | **H=12.921, P=0.002, ab*, bc*** |
| **RLS family history, n (%positive)** | 21 (35.0%) | 29 (42.0%) | 4 (18.2%) | X2=4.155, P=0.125 |
| **IRLSRS** | 23.5±7.7 | 24.2±5.6 | 25.1±4.3 | H=0.319, P=0.853 |
| **Male** | 20.2±9.0 | 22.8±6.2 | 22.8±5.9 | H=0.433, P=0.806 |
| **Female** | 23.8±7.6 | 25.3±5.0 | 25.8±3.7 | H=0.536, P=0.765 |
| **IRLSRS, severe to very severe (21-40), n (%)** | 41 (68.3%) | 53 (76.8%) | 19 (86.4%) | H=3.043, P=0.218 |
| **Severe sleep disturbance due to RLS (IRLSRS item 4≥3), n(%)** | 39 (65.0%) | 51 (73.9%) | 18 (81.8%) | X2=2.592, P=0.274 |
| **Severe tiredness or sleepiness during the day due to RLS (IRLSRS item 5≥3), n (%)** | 21 (35.0%) | 13 (18.8%) | 8 (36.4%) | X2=5.111, P=0.078 |
| **Impact on daily affairs due to RLS (IRLSRS item 9≥3), n (%))** | 8 (13.3%) | 9 (13.0%) | 1 (4.5%) | X2=1.123, P=0.656 |
| **Severe mood disturbance due to RLS (IRLSRS item 10≥3), n (%)** | 14 (23.3%) | 13 (18.8%) | 5 (22.7%) | X2=0.513, P=0.826 |
| **Chronic-persistent RLS, n (%)** | 50 (83.3%) | 63 (91.3%) | 22 (100%) | X2=4.902, P=0.077 |
| **Unilateral or unilateral dominant of RLS, n (%)** | 25 (41.7%) | 25 (36.2%) | 8 (36.4%) | X2=0.446, P=0.800 |
| **Strictly unilateral RLS, n (%)** | 2 (3.3%) | 4 (5.8%) | 2 (9.1%) | X2=1.415, P=0.483 |
| **Extra body parts involvement beyond legs, n (%)** | 13 (21.7%) | 7 (10.1%) | 4 (18.2%) | X2=3.405, P=0.175 |
| **Seasonal fluctuation, n (%)** | 20 (33.3%) | 27 (39.1%) | 8 (36.4%) | X2=0.466, P=0.792 |
| **with worsening in summer, n (%)** | 10 (16.7%) | 14 (20.3%) | 4 (18.2%) | X2=0.318, P=0.92 |
| **Haemoglobin (g/L)** | 133.8±12.3 | 143.0±12.9 | 97.6±13.4 | **H=63.677,P<0.0001, ab*,ac*,bc*** |
| **male** | 151.8±15.4 | 152.9±9.7 | 114.4±14.1 | **H=12.213,** **P=0.002, ac, bc*** |
| **female** | 132.1±10.8 | 135.9±9.9 | 92.7±8.6 | **H=45.400, P<0.0001, ac*, bc*** |
| **Ferritin (μg/L)** | 36.4±20.9* | 198.9±110.8 | 9.1±11.9* | **H=117.814,P<0.0001, ab*, ac*, bc*** |
| **male** | 53.4±17.4* | 252.0±111.9 | 23.6±18.8 | **H=20.310, P<0.0001, ab*, bc*** |
| **female** | 35.1±20.7* | 160.5±93.7 | 4.5±1.3* | **H=88.831, P<0.0001, ab*, ac*, bc*** |
| **Transferrin (g/L)** | 2.8±0.5 | 2.6±0.3 | 3.4±0.6 | **H=32.107, P<0.0001, ab, ac*, bc*** |
| **male** | 2.6±0.3 | 2.5±0.4 | 3.0±0.5 | H=3.762, P=0.152 |
| **female** | 2.8±0.6 | 2.6±0.3 | 3.5±0.6 | **H=27.655, P<0.0001, ac*, bc*** |
| **Iron (μmol/L)** | 16.1±6.4 | 19.0±5.2 | 6.0±4.6 | **H=49.069, P<0.0001, ab*, ac*, bc*** |
| **male** | 16.3±4.2 | 21.3±5.8 | 10.2±6.3 | **H=10.94, P=0.004, bc*** |
| **female** | 16.1±6.6 | 17.4±3.9 | 4.8±3.3 | **H=38.936, P<0.0001, ac*, bc*** |
| **TIBC (μmol/L)** | 60.4±9.3 | 57.0±6.5 | 73.5±10.2 | **H=33.738, P<0.0001, ac*, bc*** |
| **male** | 57.2±3.9 | 56.2±6.1 | 68.6±11.4 | H=4.417, P=0.11 |
| **female** | 60.6±9.6 | 57.7±6.7 | 75.0±9.8 | **H=28.018, P<0.0001, ac*, bc*** |

Kruskal-Wallis test for comparison of continuous variables, post hoc Mann-Whitney when p <0.05, Pearson Chi-Square or Fisher's exact test for categorical variables. *Iron deficiency was defined as ferritin level<75 μg/L, or ferritin≥75* *μg/L, however TSAT <20%.*

*Abbreviations:* *IDA: Iron deficiency anemia; IDNA: Iron deficiency without anemia; IRLSRS:* *International Restless Legs Syndrome Rating Scale; NID: non-iron deficient; TIBC: total iron-binding capacity*

**Values of serum ferritin ≥75 μg/L (all with TSAT<20%) were excluded for statistics in the IDNA (n=2) and IDA group (n=1) since these ferritin values may be ostensible due to inflammatory or other conditions.*

*ac: IDNA vs IDA at p<0.05*

*ac*: IDNA vs IDA at p<0.01*

*bc: NID vs IDA at p<0.05*

*bc*: NID vs IDA at p<0.01*

*ab: IDNA vs NID at p<0.05*

*ab*: IDNA vs NID at p<0.01*
