# Supplementary material for: Correlates of Nonanemic Iron Deficiency in Restless Legs Syndrome
Source: Front Neurol. 2020 Apr 30;11:298. doi: 10.3389/fneur.2020.00298 (PMC7205016; doi:10.3389/fneur.2020.00298)
Supplement: Supplementary file 2 [file Table_2.DOCX]

**Supplementary table 2: Demographic information, clinical features and laboratory tests in RLS patients with IDNA, NID and IDA at cut-off value of 50μg/L on ferritin for iron deficiency**

|  | **RLS with IDNA**  **(n=65)** | **RLS with NID**  **(n=131)** | **RLS with IDA**  **(n=26)** | **P value** |
| --- | --- | --- | --- | --- |
| **Female, n (%)** | 61 (93.8%) | 69 (52.7%) | 19 (73.1%) | **X2=33.846, P<0.0001, ab*, ac*** |
| **Age (yr)** | 47.6±14.1 | 55.6±12.5 | 51.2±13.8 | **H=19.453, P<0.0001, ab**,* bc** |
| **male** | 54.8±10.5 | 53.7±12.0 | 61.9±10.1 | H=3.622, P=0.163 |
| **female** | 47.2±14.2 | 57.3±12.8 | 47.3±13.0 | **H=24.004, P<0.0001, ab*, bc*** |
| **Hyperlipidemia, n (%)** | 12 (18.5%) | 29 (22.1%) | 2 (7.7%) | X2=2.947, P=0.229 |
| **Hypertension, n (%)** | 8 (12.3%) | 23 (17.6%) | 3 (11.5%) | X2=1.044, P=0.608 |
| **Diabetes, n (%)** | 3 (4.6%) | 11 (8.4%) | 5(19.2%) | X2=4.607, P=0.085 |
| **Anemia, n (%)** | 0 (0%) | 0 (0%) | 26 (100%) | **X2=149.326, P<0.0001, ac*, bc*** |
| **Age at RLS onset (yr)** | 33.5±15.8 | 39.9± 15.0 | 44.1±16.8 | **H=11.123, P=0.004, ab*,ac*** |
| **male** | 49.0±11.7 | 40.7± 14.2 | 59.0±10.1 | **H=11.103, P=0.004, bc*** |
| **female** | 32.5±15.5 | 39.1± 15.8 | 38.6±15.5 | **H=7.267, P=0.026,** **ab** |
| **Duration of RLS (yr)** | 14.1±10.7 | 15.9± 12.5 | 7.1±7.0 | **H=13.680, P=0.001, ac*, bc*** |
| **male** | 5.6±2.5 | 13.2± 10.3 | 2.9±1.1 | **H=10.540, P=0.005, bc*** |
| **female** | 14.7±10.8 | 18.2± 13.8 | 8.6±7.6 | **H=8.774, P=0.012, ac, bc*** |
| **RLS family history, n (%positive)** | 21 (32.3%) | 44 (33.6%) | 6 (23.1%) | X2=1.106, P=0.575 |
| **IRLSRS** | 23.9±8.1 | 24.2±6.5 | 25.7±6.8 | H=0.758, P=0.684 |
| **Male** | 22.3±9.4 | 23.4±6.9 | 23.9±5.1 | H=0.114, P=0.945 |
| **Female** | 24.0±8.1 | 25.0±6.1 | 26.4±3.8 | H=0.399, P=0.819 |
| **Severe sleep disturbance due to RLS (IRLSRS item 4≥3), n (%)** | 43 (66.2%) | 92 (70.2%) | 22 (84.6%) | X2=3.094, P=0.213 |
| **Severe tiredness or sleepiness during the day due to RLS (IRLSRS item 5≥3), n (%)** | 21 (32.3%) | 26 (19.8%) | 8 (30.8%) | X2=4.187, P=0.127 |
| **Impact on daily affairs due to RLS (IRLSRS item 9≥3), n (%)** | 10 (15.4%) | 17 (13.0%) | 3 (11.5%) | X2=0.327, P=0.844 |
| **Severe mood disturbance due to RLS (IRLSRS item 10≥3), n (%)** | 18 (27.7%) | 29 (22.1%) | 7 (26.9%) | X2=0.836, P=0.658 |
| **Chronic-persistent RLS, n (%)** | 56 (86.2%) | 122 (93.1%) | 26 (100%) | X2=4.938, P=0.064 |
| **Unilateral or unilateral dominant of RLS, n (%)** | 29 (44.6%) | 51 (38.9%) | 9 (34.6%) | X2=0.952, P=0.621 |
| **Strictly unilateral RLS, n (%)** | 1 (1.5%) | 10 (7.6%) | 2 (7.7%) | X2=3.357, P=0.165 |
| **Extra body parts involvement beyond legs, n (%)** | 16 (24.6%) | 25 (19.1%) | 4 (15.4%) | X2=1.257, P=0.533 |
| **Seasonal fluctuation, n (%)** | 22 (33.8%) | 43 (32.8%) | 9 (34.6%) | X2=0.979, P=1.000 |
| **with worsening in summer, n (%)** | 11 (16.9%) | 19 (14.5%) | 4 (15.4%) | X2=0.295, P=0.893 |
| **Haemoglobin (g/L)** | 134.5±13.3 | 142.9±13.7 | 97.2±12.6 | **H=77.688,P<0.0001, ab*,ac*,bc*** |
| **male** | 162.0±6.4 | 152.7±10.5 | 109.3±14.5 | **H=20.992, P<0.0001, ac*, bc*** |
| **female** | 132.7±11.6 | 134.1±9.8 | 92.7±8.5 | **H=50.443, P<0.0001, ac*, bc*** |
| **Ferritin (μg/L)** | 30.4±18.0* | 197.7±137.0 | 8.8±11.2* | **H=156.845,P<0.0001, ab*, ac*, bc*** |
| **male** | 41.8±9.5* | 264.3±148.9 | 21.4±17.7* | **H=23.292, P<0.0001, ab*, bc*** |
| **female** | 29.5±17.6*** | 139.7±93.4 | 4.6±1.4* | **H=114.648, P<0.0001, ab*, ac*, bc*** |
| **Transferrin (g/L)** | 2.9±0.5 | 2.5±0.3 | 3.3±0.6 | **H=49.672, P<0.0001, ab*, ac*, bc*** |
| **male** | 2.7±0.1 | 2.5±0.4 | 2.9±0.6 | **H=6.013, P=0.049, bc** |
| **female** | 2.9±0.5 | 2.5±0.3 | 3.5±0.5 | **H=41.217, P<0.0001, ab*, ac*, bc*** |
| **Iron (μmol/L)** | 15.0±6.3 | 18.5±5.4 | 6.0±4.3 | **H=61.990, P<0.0001, ab*, ac*, bc*** |
| **male** | 16.0±4.3 | 19.8±6. | 9.2±5.5 | **H=13.447, P=0.001, bc*** |
| **female** | 15.5±6.6 | 17.0±3.9 | 4.8±3.2 | **H=46.628, P<0.0001, ab, ac*, bc*** |
| **TIBC (μmol/L)** | 62.8±9.1 | 56.4±6.5 | 72.4±10.9 | **H=50.115, P<0.0001, ab*, ac*, bc*** |
| **male** | 59.9±1.6 | 55.4±6.6 | 65.8±12.8 | H=5.924, P=0.052 |
| **female** | 62.0±9.1 | 56.5±6.6 | 74.8±9.4 | **H=40.952, P<0.0001, ab*, ac*, bc*** |

Kruskal-Wallis test for comparison of continuous variables, post hoc Mann-Whitney when p <0.05, Pearson Chi-Square or Fisher's exact test for categorical variables. *Iron deficiency was defined as ferritin level<50 μg/L, or ferritin≥50 μg/L, however TSAT <20%.*

*Abbreviations:* *IDA: Iron deficiency anemia; IDNA: Iron deficiency without anemia; IRLSRS:* *International Restless Legs Syndrome Rating Scale; NID: non-iron deficient; TIBC: total iron-binding capacity*

**Values of serum ferritin ≥75 μg/L (all with TSAT<20%) were excluded for statistics in the IDNA (n=3) and IDA group (n=2) since these ferritin values may be ostensible due to inflammatory or other conditions.*

*ac: IDNA vs IDA at p<0.05*

*ac*: IDNA vs IDA at p<0.01*

*bc: NID vs IDA at p<0.05*

*bc*: NID vs IDA at p<0.01*

*ab: IDNA vs NID at p<0.05*

*ab*: IDNA vs NID at p<0.01*
